# Respiratory symptoms and outcomes among cigar smokers: findings from the Population Assessment of Tobacco and Health (PATH) study waves 2–5 (2014–2019)

**DOI:** 10.1186/s12931-024-02818-x

**Published:** 2024-04-27

**Authors:** Eva Sharma, Kristin Lauten, Katarzyna A. Zebrak, Kathryn C. Edwards, Samantha VanEtten, Adam F. Benson, Cristine D. Delnevo, Daniela Marshall, Heather L. Kimmel, Kristie A. Taylor, Maansi Bansal-Travers, Andrew Hyland, K. Michael Cummings

**Affiliations:** 1 Westat, 1600 Research Blvd., Rockville, MD 20850 USA; 2 Center for Tobacco Products, US Food and Drug Administration, Silver Spring, MD USA; 3 Rutgers Institute for Nicotine & Tobacco Studies, Rutgers Biomedical and Health Sciences, Rutgers University, New Brunswick, NJ USA; 4 National Institute On Drug Abuse, National Institutes of Health, Bethesda, MD USA; 5 Axle Informatics, Rockville, MD USA; 6 Department of Health Behavior, Division of Cancer Prevention & Population Sciences, Roswell Park Comprehensive Cancer Center, Buffalo, NY USA; 7 Department of Psychiatry and Behavioral Sciences, Medical University of South Carolina, Charleston, SC USA

## Abstract

**Background:**

The mechanisms by which cigarette smoking increases the risk of respiratory disease have been studied. However, less is known about risks of respiratory symptoms and outcomes associated with smoking cigars, and risks by cigar types have not been previously explored. The aim of this study was to examine associations between cigar use, including traditional cigars, cigarillos, filtered cigars, and dual cigar and cigarette use, and functionally important respiratory symptoms (FIRS), lifetime asthma diagnosis, uncontrolled asthma, and new cases of FIRS.

**Methods:**

Data from Waves 2–5 (2014–19) of the Population Assessment of Tobacco and Health (PATH) Study, a nationally representative longitudinal study, were analyzed in two ways. For cross-sectional analysis, the analytic sample included adults 18 and older at each wave, resulting in 44,040 observations. Separately, longitudinal analyses were assessed among adults 18 and older at Wave 2, resulting in 7,930 individuals. Both analyses excluded adults with chronic obstructive pulmonary disease (COPD) or non-asthma respiratory disease.

**Results:**

Current established cigarillo smokers had higher odds of having FIRS (Adjusted odds ratio (AOR): 1.72; 95% CI: 1.08, 2.74) compared to never smokers of cigarillos and cigarettes, after adjusting for covariates. Current established filtered cigar smokers had higher odds of asthma diagnosis (AOR: 1.35; 95% CI: 1.10, 1.66) while current established dual smokers of filtered cigars and cigarettes had higher odds of uncontrolled asthma (AOR: 5.13; 95% CI: 1.75, 15.02) compared to never smokers of filtered cigars or cigarettes. Both current established cigar smokers and current established dual smokers of cigarettes and cigars had higher odds of new FIRS compared to never cigar or cigarette smokers (AORs: 1.62; 95% CI: 1.02, 2.60 for exclusive cigars and 2.55; 95% CI 1.57, 4.14 for dual smokers).

**Conclusions:**

This study provides evidence that cigar smokers or dual smokers of cigars and cigarettes have greater odds of FIRS, asthma, and uncontrolled asthma and that new incidence of FIRS is higher among any cigar smokers compared to never cigar or cigarette smokers. Understanding health impacts associated with cigar use provides information for supporting policy development, as well as for designing clinical interventions focused on smoking cessation for cigars.

**Supplementary Information:**

The online version contains supplementary material available at 10.1186/s12931-024-02818-x.

## Introduction

Upwards of 87% of all respiratory disease deaths are attributable to cigarette smoking [1]. The recent National Academies of Sciences, Engineering, and Medicine (NASEM) report provided conclusive evidence that the addictive, toxic, and carcinogenic constituents of cigar tobacco are the same as cigarette tobacco and that toxicants and carcinogens in cigar smoke are qualitatively the same as those in cigarette smoke [2]. If cigar smokers are exposed to harmful constituents similar to cigarette smoke, then the risk of respiratory disease from smoking cigars could be similar to that from smoking cigarettes if frequency of use and inhalation patterns are similar. However, patterns of cigar use frequency have been shown to vary by cigar type and limited data are available regarding inhalation patterns [2].

Wheezing and coughing are some of the most prevalent respiratory symptoms that are associated with cigarette smoking, alongside conditions such as asthma [3, 4]. Wheezing is a respiratory symptom characterized by a high-pitched whistling sound during expiration or inspiration due to airway obstruction, [5] which is suggestive of asthma and/or chronic bronchitis [6]. Asthma, another highly prevalent respiratory outcome, is characterized as a disease of reversible expiratory airflow limitation [7]. While these symptoms and outcomes have been extensively studied among cigarette smokers, there have been very few studies on other tobacco products such as cigars, and the ones that examined cigars have been inconclusive. In a cross-sectional study using Population Assessment of Tobacco and Health (PATH) Study Wave 3 (2015/16) adult data, Schneller and colleagues found higher odds of ever wheezing among current cigarette and electronic nicotine delivery system (ENDS) users compared to noncurrent cigarette and ENDS users but not among cigar smokers [8]. Using PATH Study Wave 2 (2014/15) and Wave 3 (2015/16) adult data, Sargent and colleagues examined longitudinal associations between functionally-important respiratory symptoms (FIRS; scores ≥ 3 on FIRS index are indicative of respiratory symptoms and can be used to assess relationships between tobacco and respiratory health) and cigar use and did not find any association between exclusive cigar use and worsening of respiratory symptoms [9]. Conversely, other studies have found a link between cigar use and respiratory symptoms. In a cross-sectional study among adults aged 45–84 years from six U.S. communities, Rodriguez et al. found decrements in lung function and increased odds of airflow obstruction among cigar smokers who had never smoked cigarettes, suggesting lung damage among long-term cigar smokers [10]. In a nationally representative study using Wave 2 (2014/15) PATH Study data from 10,267 adults aged 18–39 without chronic obstructive pulmonary disease (COPD), Brunette and colleagues found a significant relationship between lifetime asthma diagnosis and current cigar use. [11] Together, these studies suggest that long-term and short-term findings related to cigar use and respiratory outcomes have been mixed and the effects of cigar use on these outcomes remain unclear. In addition, most of the literature on cigar use is not specific to cigar type such as traditional cigars, cigarillos, and filtered cigars, which differ in shape, size, manufacturing process, packaging size, price, user characteristics, and patterns of use, and other qualities [12]. To our knowledge, no study to date has examined respiratory outcomes by cigar type.

In this study, we used Waves 2 (2014–2015) to 5 (2018–2019) of the PATH Study data to examine associations between the use of traditional cigars, cigarillos, and filtered cigars and respiratory symptoms and outcomes such as FIRS, lifetime asthma diagnosis, and uncontrolled asthma. The PATH Study data allowed exploration of respiratory outcomes by cigar type as the type may affect the risks of respiratory outcomes. We hypothesized that adults who smoke any type of cigar have a higher risk of respiratory conditions compared to never users of cigars and cigarettes. Given any cigar use has remained stable over recent years while cigarette smoking has decreased, understanding health outcomes associated with cigar use is important for public health [13–15].

## Methods

The PATH Study is an ongoing, nationally representative, longitudinal cohort study of adults and youth in the U.S. that collects information on tobacco-use patterns and associated health behaviors. The PATH Study recruitment for the Wave 1 Cohort employed a stratified address-based, area-probability sampling design at Wave 1 (2013) that oversampled adult tobacco users, young adults (aged 18–24), and African American adults. Differences in the number of completed interviews between Wave 1 and subsequent waves reflect attrition (e.g., nonresponse, mortality). The total unweighted attrition rate among the Wave 1 sample was 16% at Wave 2, 21% at Wave 3, 27% at Wave 4, and 30% at Wave 5. Full-sample and replicate weights were created to adjust for the complex sample design (e.g., oversampling of particular demographic groups) and attrition. Further details regarding the PATH Study design and methods for the Wave 1 Cohort are published elsewhere [16–18]. Details on interview procedures, questionnaires, sampling, weighting, response rates, and accessing the data are described in the PATH Study Restricted Use Files (RUF) User Guide at https://doi.org/10.3886/Series606. The study was conducted by Westat and approved by the Westat Institutional Review Board. All respondents aged 18 and older provided informed consent.

**Fig. 1:**
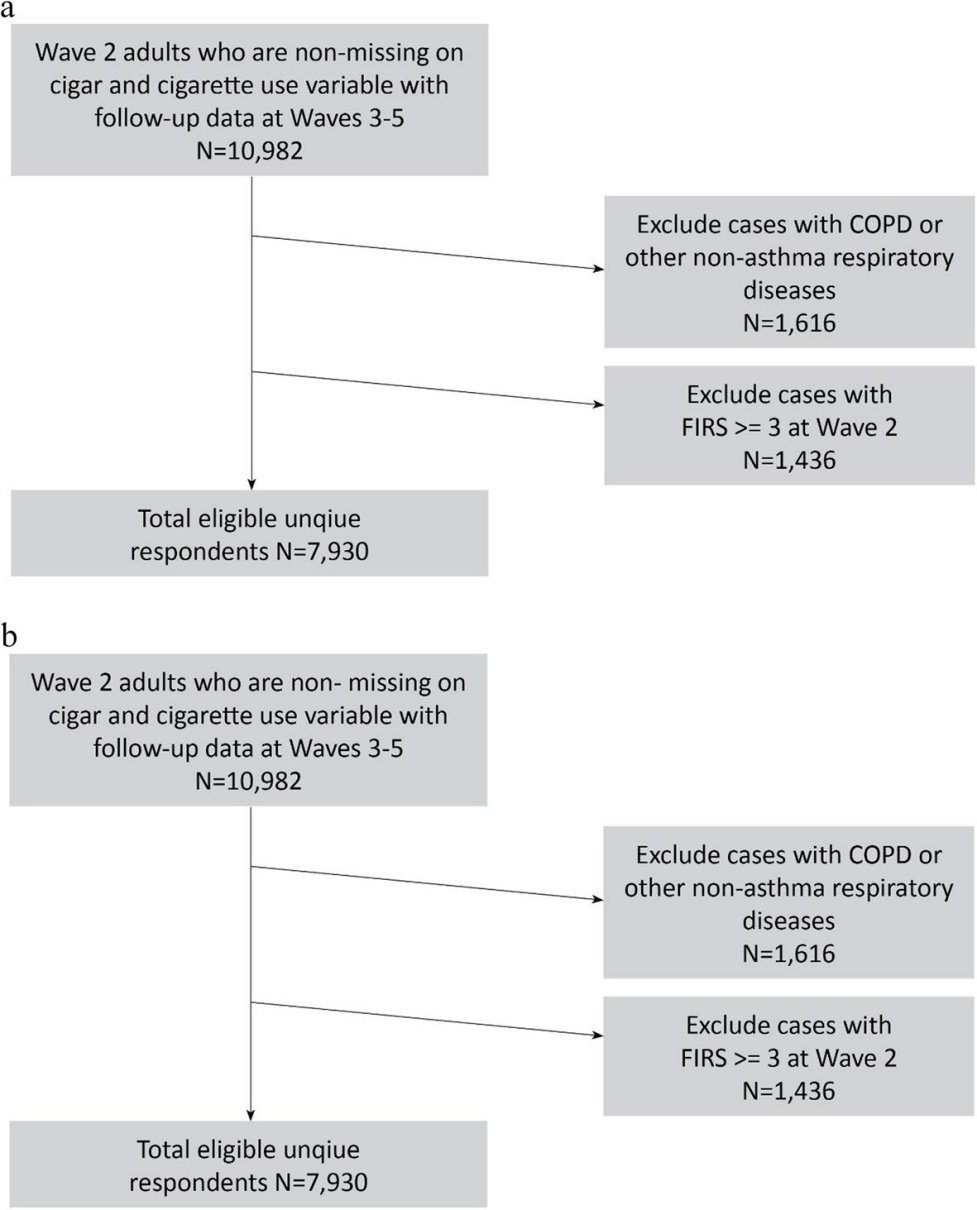
**a** Waves 2–5 analytic sample from the Wave 1 Cohort for cross-sectional analysis using weighted GEE methods. Note: The N increases at each wave due to the aging up of youth into the adult sample. **b**. Waves 2–5 analytic sample from the Wave 1 Cohort to examine new cases of FIRS

This study uses the PATH Study RUF data files and the Wave 5 all-waves weights for the Wave 1 Cohort to analyze four waves of data: Wave 2 (2014–2015), Wave 3 (2015–2016), Wave 4 (2016–2018) and Wave 5 (2018–2019) from adult respondents 18 years and older. The interview interval for Waves 2–4 was approximately one year, while the interval for Waves 4–5 was approximately two years. Wave 1 data were not included because most respiratory symptoms were not assessed until Wave 2. The current study analyzes data in two ways: 1) examines cross-sectional (Waves 2–5) associations between cigar and cigarette use status and FIRS, asthma, and uncontrolled asthma, restricted to adults age 18 and older at Waves 2–5 (total N_obs_ = 44,040; Fig. 1a); and 2) examines longitudinal associations between cigar and cigarette use status and new cases of FIRS during the period of Waves 3–5 among Wave 2 adults 18 and older who did not have FIRS at Wave 2 (*N* = 7,930 unique respondents; Fig. 1b). All analyses excluded respondents with pre-existing COPD or other non-asthma respiratory diseases. In addition, for both approaches, analyses were limited to study participants who were interviewed at every wave. The study also intended to examine incidence of asthma related outcomes but was not sufficiently powered to do so.

## Measures

### Respiratory outcomes

#### FIRS

As described by Halenar et al., [19] three wheezing variables (ever wheezing, past 12-month (P12M) wheezing, and P12M wheezing attack frequency) were combined to create one 5-level variable (0 = never wheezing; 1 = ever, but no P12M wheezing OR P12M wheezing but no wheezing attacks; 2 = P12M wheezing and 1–3 attacks; 3 = P12M wheezing and 4–12 attacks; 4 = P12M wheezing and more than 12 attacks). The score from this variable was summed with scores from four additional questions (see Table 7 in Appendix) to create the index ranging from 0 to 9, where 0 represented no respiratory symptoms and 9 represented the highest level of symptoms. This index was dichotomized based on the indication that a cut-off value of ≥ 3 symptoms had high sensitivity (~ 68%) for detecting those with asthma using asthma medication(s), and was predictive of functional limitations (i.e., difficulty walking one mile) [19]. For the incidence analysis, only participants with FIRS ≥ 3 at baseline were excluded; i.e., those who had 0–2 respiratory symptoms were included to examine incidence of significant FIRS. See Table 7 in Appendix for details.

#### Asthma diagnosis

Lifetime asthma diagnosis was indicated if a respondent selected “asthma” for the following question, “Has a doctor, nurse or other health professional ever told you that you had any of the following lung or respiratory conditions?” Options included COPD, Chronic Bronchitis, Emphysema, Asthma, some other lung or respiratory condition, None of the above.

Asthma control test (ACT): The ACT is a validated five-item questionnaire designed for asthma patients to self-report asthma symptoms, each item on a scale from 1–5 [11]. Items included impact of asthma on daily activities, frequency of shortness of breath, sleep disturbance due to asthma symptoms, frequency of use of asthma controlling medications, and self-rating of asthma control (see Table 7 in Appendix). The time of reference for all items was within the past 30 days. Scores from each of the five items were combined into a summary score that ranged from 5–25 (higher = better asthma control, lower = more symptoms). An ACT score of 19 had high sensitivity (71.3%) and specificity (70.8%) for detecting uncontrolled asthma in nonsmokers and a score of 18.6 provided the maximum area under the receiver operator characteristic (ROC) curve in cigarette smokers [11]. Uncontrolled asthma was defined as having a score of 19 or less [11].

#### Tobacco use

At each wave, respondents were asked about current use (every day, some days, not at all) of three cigar types: traditional cigars, cigarillos, and filtered cigars. Responses from each cigar type were combined to represent “any cigar” smoking. Current established any cigar smoking was defined as ever smoking any cigar fairly regularly and now smoking every day or some days. At each wave, current established cigarette smoking was defined as having smoked at least 100 cigarettes in one’s lifetime and now smoking every day or some days. A four-category variable was created as follows: 1) never smokers of any cigars or cigarettes (could use other tobacco products), 2) current established any cigar smokers (no current established cigarette use and could use other tobacco products), 3) current established cigarette smokers (no current established cigar use and could use other tobacco products), and 4) dual current established smokers of any cigar and cigarettes (could be users of other tobacco products). The same logic was applied for individual cigar type variables (See Table 7 in Appendix for details). To account for use of other tobacco products, a variable that represented current established use of ENDS, pipe, hookah, smokeless tobacco, or snus was created.

#### Covariates

Covariates in the analyses included age (18–24, 25–39, 40–54, 55 + , and collapsed 18–39 vs. 40 + for asthma diagnosis and uncontrolled asthma), sex, race/ethnicity, education, past 30-day marijuana use, body mass index (BMI) based on self-reported height and weight, cigarette pack years, [19] duration of cigar use measured in years, second hand exposure measured in hours, and use of asthma medications among those with asthma (in models exploring uncontrolled asthma).

### Statistical analyses

**Fig. 2:**
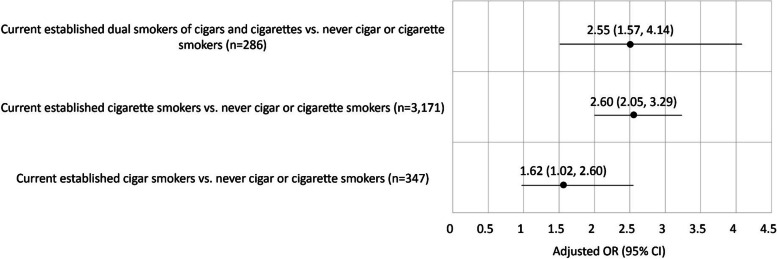
Associations between any cigar and cigarette use status^1^ at Wave 2 and new reports of ≥ 3 functionally important respiratory symptoms (*N* = 7,210^2^) at Wave 3, Wave 4, or Wave 5, PATH Study 2014–2019. OR = odds ratio. ORs are weighted, Ns are unweighted using the Wave 5 all-waves weights for the Wave 1 Cohort. Excludes those with COPD and other non-asthma respiratory diseases. ^1^Current established any cigar smoking was defined as ever smoking any cigar fairly regularly and now smoking every day or some days. ^2^Ns are based on unadjusted OR sample size and do not take into account missingness on covariates. 583 observations were missing on covariates in the adjusted model. N for never cigar or cigarette smokers = 3,406. Adjusted for age (18–24, 25–39, 40–54, 55 +), sex, race/ethnicity, education, BMI, cigarette pack years, duration of cigar use, secondhand smoke exposure, past month marijuana use, and current established use of at least one of: ENDS, pipe, hookah, smokeless tobacco, snus

The current study examined associations between cigar use and respiratory symptoms and outcomes using four waves of the PATH Study (2014–19) data in two ways, cross-sectional and longitudinal:

1. For cross-sectional analysis, the prevalence of FIRS, asthma, and uncontrolled asthma was examined by cigar and cigarette use status, using four waves (Waves 2–5) of within-person design using repeated measures, controlling for age, sex, race/ethnicity, education level, current established use of other tobacco products, body mass index (BMI), marijuana use, cigarette pack years, secondhand smoke exposure, and duration of cigar use. Weighted generalized estimating equations (GEEs) were used (Fig. 1a explains how samples were drawn) to evaluate the user group differences in respiratory symptoms (Tables 1, 2 and 3) and outcomes (Tables 4, 5 and 6) to obtain statistically valid population estimates and test statistics. The GEE method produces population-averaged estimates [20] and allows for inclusion of multiple waves of data in a single analysis while statistically controlling for interdependence among observations contributed by the same individuals [21, 22]. Specifically, GEE logistic regression models used within-person autoregressive correlation structure and the binomial distribution of the dependent variable using the logit link function.
2. For longitudinal analysis, weighted odds ratios (ORs) were obtained from logistic regression models to examine the unadjusted and adjusted longitudinal associations of different types of cigar use at Wave 2 with incident FIRS during the follow-up periods of Waves 3–5 (Fig. 2 and Supplemental Table 2), controlling for covariates at Wave 2. Figure 1b explains how samples were drawn.

**Table 1:** Associations between traditional cigar and cigarette use status and functionally important respiratory symptoms (Wave 2—Wave 5 population averaged), PATH Study 2014–2019

Tobacco use status	**Functionally important respiratory symptoms** **(≥ 3 symptoms) Obs = 41,112** ^a^			
	Obs	Weighted % (95% CI)	Unadjusted OR (95% CI)	Adjusted OR (95% CI)
Never smokers of cigarettes or traditional cigars Obs = 19,333	1,289	6.0 (5.3, 6.7)	**Ref**	**Ref**
Current established traditional cigar smokers Obs = 1,034	94	7.3 (5.4, 9.7)	1.43 (0.98, 2.08)	0.82 (0.52, 1.28)
Current established cigarette smokers Obs = 20,232	4,693	22.3 (21.3, 23.3)	**4.93 (4.25, 5.72)**	**2.49 (2.08, 2.97)**
Current established dual smokers of traditional cigars and cigarettes Obs = 513	120	23.2 (17.9, 29.4)	**4.65 (3.43, 6.31)**	**2.40 (1.64, 3.51)**

*OR *Odds ratio, *Obs *Observations

Bolded estimates are statistically significant (*p* < 0.05)

Ns are unweighted; percentages and ORs are weighted using the Wave 5 all-waves weights for the Wave 1 Cohort

Excludes those with COPD and other non-asthma respiratory diseases

Adjusted for age (18–24, 25–39, 40–54, 55 +), sex, race/ethnicity, education, BMI, cigarette pack years, duration of cigar use, secondhand smoke exposure, past month marijuana use, and current established use of at least one of: ENDS, pipe, hookah, smokeless tobacco, snus. Also adjusted for current established use of cigarillos and filtered cigars

^a^Overall Obs represents unadjusted model and does not take into account missingness on covariates. 11,031 observations were missing on covariates in the adjusted model

**Table 2:** Associations between cigarillo and cigarette use status and functionally important respiratory symptoms (Wave 2—Wave 5 population averaged), PATH Study 2014–2019

Tobacco use status	**Functionally important respiratory symptoms (≥ 3 symptoms) Obs = 40,897** ^a^			
	Obs	Weighted % (95% CI)	Unadjusted OR (95% CI)	Adjusted OR (95% CI)
Never smokers of cigarettes or cigarillos Obs = 19,296	1,283	6.0 (5.3, 6.7)	**Ref**	**Ref**
Current established cigarillo smokers Obs = 890	98	11.0 (8.3, 14.5)	**2.98 (1.98, 4.49)**	**1.72 (1.08, 2.74)**
Current established cigarette smokers Obs = 19,657	4,544	22.2 (21.2, 23.2)	**4.92 (4.23, 5.72)**	**2.53 (2.11, 3.03)**
Current established dual smokers of cigarillos and cigarettes Obs = 1,054	258	24.9 (21.2, 29.0)	**5.40 (4.12, 7.09)**	**2.95 (2.13, 4.07)**

*OR* odds ratio, *Obs* observations

Bolded estimates are statistically significant (*p* < 0.05)

Ns are unweighted; percentages and ORs are weighted using the Wave 5 all-waves weights for the Wave 1 Cohort

Excludes those with COPD and other non-asthma respiratory diseases

Adjusted for age (18–24, 25–39, 40–54, 55 +), sex, race/ethnicity, education, BMI, cigarette pack years, duration of cigar use, secondhand smoke exposure, past month marijuana use, and current established use of at least one of: ENDS, pipe, hookah, smokeless tobacco, snus. Also adjusted for current established use of traditional cigars and filtered cigars

^a^Overall Obs represents unadjusted model and does not take into account missingness on covariates. 11,044 observations were missing on covariates in the adjusted model

**Table 3:** Associations between filtered cigar and cigarette use status and functionally important respiratory symptoms (Wave 2 – Wave 5 population averaged), PATH Study 2014–2019

Tobacco use status	**Functionally important respiratory symptoms (≥ 3 symptoms) Obs = 40,446** ^a^			
	Obs	Weighted % (95% CI)	Unadjusted OR (95% CI)	Adjusted OR (95% CI)
Never smokers of cigarettes or filtered cigars Obs = 19,406	1,289	5.9 (5.3, 6.7)	**Ref**	**Ref**
Current established filtered cigar smokers Obs = 298	49	14.7 (10.4, 20.3)	**4.28 (2.70, 6.80)**	**2.36 (1.32, 4.20)**
Current established cigarette smokers Obs = 20,115	4,659	22.3 (21.3, 23.3)	**4.96 (4.27, 5.76)**	**2.57 (2.15, 3.08)**
Current established dual smokers of filtered cigars and cigarettes Obs = 627	153	23.2 (19.2, 27.7)	**4.77 (3.55, 6.41)**	**2.39 (1.69, 3.38)**

*OR* odds ratio, *Obs* observations

Bolded estimates are statistically significant (*p* < 0.05)

Ns are unweighted; percentages and ORs are weighted using the Wave 5 all-waves weights for the Wave 1 Cohort

Excludes those with COPD and other non-asthma respiratory diseases

Adjusted for age (18–24, 25–39, 40–54, 55 +), sex, race/ethnicity, education, BMI, cigarette pack years, duration of cigar use, secondhand smoke exposure, past month marijuana use, and current established use of at least one of: ENDS, pipe, hookah, smokeless tobacco, snus. Also adjusted for current established use of traditional cigars and cigarillos

^a^Overall Obs represents unadjusted model and does not take into account missingness on covariates. 10,847 observations were missing on covariates in the adjusted model

**Table 4:** Associations between traditional cigar and cigarette use status and lifetime asthma diagnosis and uncontrolled asthma (Wave 2 – Wave 5 population averaged), PATH Study 2014–2019

**Lifetime asthma diagnosis** **Obs = 42,871** ^**a**^					**Uncontrolled asthma** ^c^ ** (ACT ≤ 19)** **Obs = 5,700** ^a^				
Tobacco use status	Obs	Weighted % (95% CI)	Unadjusted OR (95% CI)	Adjusted OR (95% CI)	Tobacco use status	Obs	Weighted % (95% CI)	Unadjusted OR (95% CI)	Adjusted OR (95% CI)
Never smokers of cigarettes or traditional cigars Obs = 20,247	3,134	11.5 (10.5, 12.6)	**Ref**	**Ref**	Never smokers of cigarettes or traditional cigars Obs = 2,912	306	12.5 (10.1, 15.6)	**Ref**	**Ref**
Current established traditional cigar smokers Obs = 1,060	130	9.9 (7.4, 13.3)	0.94 (0.80, 1.11)	1.08 (0.85, 1.37)	Current established traditional cigar smokers Obs = 117	10	8.3^b^ (3.9, 16.8)	0.74 (0.30, 1.81)	0.74 (0.22, 2.56)
Current established cigarette smokers Obs = 21,039	2,862	11.9 (11.1, 12.8)	1.01 (0.89, 1.15)	**1.18 (1.03, 1.35)**	Current established cigarette smokers Obs = 2,591	606	23.5 (20.5, 26.8)	**2.09 (1.48, 2.95)**	**1.86 (1.17, 2.96)**
Current established dual smokers of traditional cigars and cigarettes Obs = 525	87	13.8 (9.6, 19.4)	1.03 (0.90, 1.18)	**1.20 (1.04, 1.38)**	Current established dual smokers of traditional cigars and cigarettes Obs = 80	24	37.4 (22.2, 55.5)	**2.66 (1.27, 5.55)**	2.15 (0.78, 5.97)

*OR* odds ratio, *Obs* observations

Bolded estimates are statistically significant (*p* < 0.05)

Ns are unweighted; percentages and ORs are weighted using the Wave 5 all-waves weights for the Wave 1 Cohort

Excludes those with COPD and other non-asthma respiratory diseases

Adjusted for age (18–39 vs. 40 +), sex, race/ethnicity, education, BMI, cigarette pack years, duration of cigar use, secondhand smoke exposure, past month marijuana use, and current established use of at least one of: ENDS, pipe, hookah, smokeless tobacco, snus. Also adjusted for current established use of cigarillos and filtered cigars. Uncontrolled asthma model also adjusted for use of asthma medications

^a^Overall Obs represents unadjusted model and does not take into account missingness on covariates. 9,616 observations were missing on covariates for the ever asthma adjusted model and 1,690 for the uncontrolled asthma adjusted model

^b^Estimate should be interpreted with extra caution because it has low statistical precision. It is based on a denominator sample size of less than 50, or the coefficient of variation of the estimate or its complement is larger than 30%

^c^Among adults with an asthma diagnosis

**Table 5:** Associations between cigarillo and cigarette use status and lifetime asthma diagnosis and uncontrolled asthma (Wave 2 – Wave 5 population averaged), PATH Study 2014–2019

**Lifetime asthma diagnosis** **Obs = 42,652** ^**a**^					**Uncontrolled asthma** ^b^ ** (ACT ≤ 19)** **Obs = 5,688** ^a^				
Tobacco use status	Obs	Weighted % (95% CI)	Unadjusted OR (95% CI)	Adjusted OR (95% CI)	Tobacco use status	Obs	Weighted % (95% CI)	Unadjusted OR (95% CI)	Adjusted OR (95% CI)
Never smokers of cigarettes or cigarillos Obs = 20,205	3,119	11.5 (10.4, 12.6)	**Ref**	**Ref**	Never smokers of cigarettes or cigarillos Obs = 2,898	305	12.6 (10.1, 15.6)	**Ref**	**Ref**
Current established cigarillo smokers Obs = 920	147	14.7 (11.4, 18.9)	0.97 (0.86, 1.09)	1.10 (0.96, 1.27)	Current established cigarillo smokers Obs = 129	23	18.8 (10.6, 31.2)	1.52 (0.41, 5.62)	1.88 (0.31, 11.33)
Current established cigarette smokers Obs = 20,404	2,725	11.8 (10.9, 12.6)	1.02 (0.90, 1.15)	**1.18 (1.03, 1.34)**	Current established cigarette smokers Obs = 2,465	582	23.8 (20.8, 27.0)	**2.09 (1.48, 2.95)**	**1.84 (1.15, 2.93)**
Current established dual smokers of cigarillos and cigarettes Obs = 1,123	214	15.9 (13.2, 19.1)	1.01 (0.89, 1.15)	**1.18 (1.03, 1.34)**	Current established dual smokers of cigarillos and cigarettes Obs = 196	46	26.1 (18.6, 35.3)	**2.20 (1.26, 3.83)**	1.81 (0.94, 3.48)

*OR* odds ratio, *Obs* observations

Bolded estimates are statistically significant (*p* < 0.05)

Ns are unweighted; percentages and ORs are weighted using the Wave 5 all-waves weights for the Wave 1 Cohort

Excludes those with COPD and other non-asthma respiratory diseases

Adjusted for age (18–39 vs. 40 +), sex, race/ethnicity, education, BMI, cigarette pack years, duration of cigar use, secondhand smoke exposure, past month marijuana use, and current established use of at least one of: ENDS, pipe, hookah, smokeless tobacco, snus. Also adjusted for current established use of traditional cigars and filtered cigars. Uncontrolled asthma model also adjusted for use of asthma medications

^a^Overall Obs represents unadjusted model and does not take into account missingness on covariates. 9,645 observations were missing on covariates for the ever asthma adjusted model and 1,712 for the uncontrolled asthma adjusted model

^b^Among adults with an asthma diagnosis

**Table 6:** Associations between filtered cigar and cigarette use status and lifetime asthma diagnosis and uncontrolled asthma (Wave 2 – Wave 5 population averaged), PATH Study 2014–2019

**Lifetime asthma diagnosis** **Obs = 42,195** ^**a**^					**Uncontrolled asthma** ^b^ ** (ACT ≤ 19)** **Obs = 5,656** ^a^				
Tobacco use status	Obs	Weighted % (95% CI)	Unadjusted OR (95% CI)	Adjusted OR (95% CI)	Tobacco use status	Obs	Weighted % (95% CI)	Unadjusted OR (95% CI)	Adjusted OR (95% CI)
Never smokers of cigarettes or filtered cigars *Obs* = *20,319*	3,145	11.5 (10.5, 12.6)	**Ref**	**Ref**	Never smokers of cigarettes or filtered cigars *Obs* = *2,924*	306	12.5 (10.0, 15.5)	**Ref**	**Ref**
Current established filtered cigar smokers *Obs* = *315*	70	22.0 (15.2, 30.7)	1.01 (0.78, 1.30)	**1.35 (1.10, 1.66)**	Current established filtered cigar smokers *Obs* = *62*	16	24.6 (14.8, 38.0)	2.24 (0.90, 5.57)	3.80 (0.86, 16.77)
Current established cigarette smokers *Obs* = *20,904*	2,843	11.9 (11.0, 12.8)	1.02 (0.90, 1.17)	**1.19 (1.04, 1.36)**	Current established cigarette smokers *Obs* = *2,573*	594	23.4 (20.3, 26.7)	**2.07 (1.45, 2.94)**	**1.89 (1.19, 3.01)**
Current established dual smokers of filtered cigars and cigarettes *Obs* = *657*	105	14.1 (10.6, 18.7)	1.09 (0.94, 1.26)	**1.27 (1.06, 1.52)**	Current established dual smokers of filtered cigars and cigarettes *Obs* = *97*	35	37.7 (25.4, 51.8)	**3.85 (2.07, 7.18)**	**5.13 (1.75, 15.02)**

*OR* odds ratio, *Obs* observations

Bolded estimates are statistically significant (*p* < 0.05)

Ns are unweighted; percentages and ORs are weighted using the Wave 5 all-waves weights for the Wave 1 Cohort

Excludes those with COPD and other non-asthma respiratory diseases

Adjusted for age (18–39 vs. 40 +), sex, race/ethnicity, education, BMI, cigarette pack years, duration of cigar use, secondhand smoke exposure, past month marijuana use, and current established use of at least one of: ENDS, pipe, hookah, smokeless tobacco, snus. Also adjusted for current established use of traditional cigars and cigarillos. Uncontrolled asthma model also adjusted for use of asthma medications

^a^Overall Obs represents unadjusted model and does not take into account missingness on covariates. 9,460 observations were missing on covariates for the ever asthma adjusted model and 1,686 for the uncontrolled asthma adjusted model

^b^Among adults with an asthma diagnosis

Weighted odds ratios (ORs) and 95% CI were reported for both unadjusted and adjusted models.

All analyses were weighted using Wave 5 all-waves weights for the Wave 1 Cohort (including full-sample and 100 replicate weights) to produce nationally representative estimates. These weights at Wave 5 were created for those who were interviewed at all previous waves. Variances were computed using the balanced repeated replication method [23] (BRR) with Fay’s adjustment set to 0.3 to increase estimate stability. [24] Statistical analyses were performed using Stata survey data procedures, version 17 (StataCorp LLC, College Station, TX).

## Results

Respiratory symptoms and outcomes at baseline (Wave 2) by tobacco use status are presented in Supplemental Table 1. Overall, at baseline, among adults who smoked cigars, 19.3% (95% CI: 16.4, 22.5) were current established smokers of more than one cigar type (traditional cigars, cigarillos, filtered cigars), 32.4% (95% CI: 28.6, 36.5) only smoked traditional cigars, 33.4% (95% CI: 30.1, 36.8) only smoked cigarillos, and 14.9% (95% CI: 12.4, 17.9) only smoked filtered cigars (data not shown in table).

### Associations between Waves 2–5 population-averaged prevalence of FIRS ≥ 3 and individual cigar/cigarette use

Unadjusted and adjusted odds ratios (AOR) for FIRS by individual cigar type and cigarette use status are shown in Tables 1, 2 and 3. For each individual cigar type, user groups were compared to never smokers; each cigar user group had significantly higher odds of having FIRS after adjusting for all covariates with the exception of current established traditional cigar smokers (not current established cigarette smokers) (AOR range: 1.72–2.95). The greatest odds of having FIRS were for current established dual use of cigarillos and cigarettes (AOR: 2.95; 95% CI: 2.13, 4.07).

### Associations between Waves 2–5 population-averaged prevalence of asthma and uncontrolled asthma and individual cigar/cigarette use

Unadjusted and adjusted odds ratios for lifetime asthma diagnosis and uncontrolled asthma by cigar and cigarette use status are shown in Tables 4, 5 and 6. Dual users of cigarettes and each individual cigar type had significantly higher odds of a lifetime asthma diagnosis compared to never smokers in adjusted models (AOR range 1.18–1.27). Current established filtered cigar smokers also had significantly higher odds of asthma diagnosis compared to never smokers of cigarettes or filtered cigars (Table 6; AOR: 1.35; 95% CI: 1.10, 1.66). In addition, current established dual smokers of filtered cigars and cigarettes had significantly higher odds of uncontrolled asthma compared to never smokers of cigarettes or filtered cigars (Table 6; AOR: 5.13; 95% CI: 1.75, 15.02). Due to small sample sizes, we were unable to stratify models by ages 18–39 and 40 + as recommended by Brunette et al [11]. Our findings, however, were adjusted for 18–39 and 40 + age groups.

### New cases of FIRS at Waves 3–5 among adults at Wave 2 without FIRS by any cigar/cigarette use

New cases of FIRS at Waves 3, 4 or 5 among Wave 2 adults without FIRS (*n* = 7,210 with no missing data on FIRS at Waves 3–5) are shown in Fig. 2 and Supplemental Table 2. The three cigar types were combined to represent any current established cigar use due to a low number of observations. Each user group had significantly higher odds of having new FIRS compared to never smokers of cigars or cigarettes with current established cigarette smokers (not current established cigar smokers) and current established dual smokers of cigars and cigarettes each having 2.6 times greater odds of new FIRS compared to never smokers. Due to insufficient number of observations, the new cases of asthma and uncontrolled asthma could not be examined.

## Discussion

Our study provided evidence that smoking any cigar increases the odds of developing FIRS in adults with no previous history of COPD or other non-asthma respiratory disease and the findings were robust after adjusting for potential confounders. As shown in Fig. 2, the association of dual use of cigarettes and any cigar on new cases of ≥ 3 respiratory symptoms was similar to that from cigarette use. However, we found that cigar smokers had higher odds for having respiratory symptoms compared to never smokers, although the odds were lower than those from cigarette smoking. One possible reason for this finding could be the differences in how cigarettes and cigars are inhaled. The more acidic pH of cigarette smoke is not as easily absorbed by the oral mucosa but is inhaled in the lungs, while the alkaline pH of cigar smoke allows absorption of smoke in the oral mucosa without inhaling [25]. Inhalation of smoke into the lungs provides a larger surface area for absorption, [25] which could affect risks for respiratory symptoms [26] To our knowledge, this is one of the first studies to report an increase in respiratory symptoms in users of some cigars. Future studies could examine the risks of individual cigar types that we were not able to do in this study.

FIRS were found to be cross-sectionally associated with smoking of cigarillos and filtered cigars compared to never use of cigarettes and cigarillos or never use of cigarettes and filtered cigars, respectively, among adults after adjustment for demographics, secondhand smoke exposure, cigarette smoking history, use of other tobacco products, use of other cigar types and marijuana. However, we did not observe a significant association between traditional cigar use and FIRS. This could be because compared to other cigar types, traditional cigars, that include premium and non-premium large cigars, are smoked differently, in terms of frequency of use and ultimately cumulative exposure to tobacco smoke [2]. In an earlier study, Sargent and colleagues [9] found no significant association between FIRS and exclusive cigar use where all three cigar types were combined into any cigar use. Our finding suggests that cigarillos and filtered cigars may affect wheezing and coughing differently than traditional cigars. We could not infer causality from our study and further longitudinal research may help establish causality for individual cigar types separately.

In this nationally representative sample of adults, in cross-sectional analyses, we report higher odds of lifetime asthma diagnosis among dual smokers of cigarettes and individual cigar types compared to never smokers, when excluding adults with COPD or other non-asthma respiratory illnesses. Similar to FIRS, the effect of dual use of cigarettes and any cigar on lifetime asthma diagnosis was similar to that from cigarette use. For uncontrolled asthma, we only found significant associations with cigarette smoking, which is similar to what other studies have found [27, 28]. One noteworthy finding was higher odds of uncontrolled asthma for dual users of filtered cigars and cigarettes [1] compared to never smokers of filtered cigars or cigarettes. However, we did not find any association between uncontrolled asthma and use of only filtered cigars, which could have been because of small sample size of current filtered cigar users. Future studies could further explore this finding.

Some limitations need to be acknowledged. The respiratory outcomes were based on self- reported measures rather than clinical diagnoses and the self-reported tobacco use measures could be subject to recall bias. In addition, even though asthma may be misdiagnosed as COPD in adults 40 and older, [22, 29] we could not stratify asthma outcome models among adults aged 18–39 and 40 and older because of small sample sizes. Additionally, the amount of missing data on covariates is a possible limitation. Models deleting observations due to missingness of data may lead to biased results and loss of power [30, 31]. We did, however, incorporate several methodological suggestions made by earlier studies [9, 19] and used validated outcomes such as FIRS [19] instead of using wheezing and coughing questions in isolation where an endorsement of any one item is considered symptomatic [9]. We excluded adults with pre-existing COPD as it is strongly associated with cigarette smoking and also wheezing. We also adjusted for confounders such as cigarette smoking history and concurrent marijuana use.

Despite the limitations, this study made an important contribution to our understanding of respiratory health and cigar smoking. The risks of respiratory disease due to cigarette smoking have been extensively studied, [1, 32] while less is known about the risks of respiratory outcomes associated with smoking cigars. This study attempted to understand the risks of cigar use on respiratory health which is a current research priority according to the 2022 NASEM report [33]. Continued research in this area may provide information for policy development and reinforce the need for clinical interventions to reduce cigar use in adults.

### Supplementary Information

**Additional file 1: Supplemental Table 1.** Characteristics of adults at Wave 2 (2014-2015) by respiratory symptoms and outcomes.** Supplemental Table 2a.** Associations between tobacco use status and functionally important respiratory symptoms (Wave 2 - Wave 5 population averaged), PATH Study 2014-2019.** Supplemental Table 2b.** Associations between tobacco use status and respiratory outcomes, stratified by adults ages 18-39 and 40+ (Wave 2 – Wave 5 population averaged), PATH Study 2014-2019.** Supplemental Table 3. **Associations between cigar and cigarette use status at Wave 2 and new reports of ≥ 3 functionally important respiratory symptoms at Wave 3, Wave 4, or Wave 5, PATH Study 2014-2019.

## Data Availability

Details on accessing the PATH Study data are described in the PATH Study Restricted Use Files website located at https://doi.org/10.3886/ICPSR36231.v29. Access to these data is restricted. Users interested in obtaining these data must complete a Restricted Data Use Agreement.

## Appendix

**Table 7:** Measures

Outcomes	Questions in the PATH Study	Response options/Notes
**Functionally important respiratory symptoms (FIRS)**	Have you ever had wheezing or whistling in the chest at any time in the past?	Yes/No
	Have you had wheezing or whistling in the chest in the past 12 months?	Yes/No
	How many attacks of wheezing have you had in the past 12 months?	None, 1–3, 4–12, more than 12
	In the past 12 months, how often, on average has your sleep been disturbed due to wheezing?	None, less than one night/week, one or more nights/week
	In the past 12 months, has wheezing ever been severe enough to limit your speech to only one or two words between breaths?	Yes/No
	In the past 12 months, has your chest sounded wheezy during or after exercise?	Yes/No
	In the past 12 months, have you had a dry cough at night, apart from a cough associated with a cold or chest infection?	Yes/No
**Functionally important respiratory symptoms (FIRS) > = 3 symptoms**	Adults who have at least 3 functionally important respiratory symptoms	Yes/No
**Asthma Control Test (ACT)**	In the past 30 days, how much of the time did your asthma keep you from getting as much done at work, school or at home?	1. All of the time 2. Most of the time 3. Some of the time 4. A little of the time 5. None of the time
	In the past 30 days, how often have you had shortness of breath?	1. More than once a day 2. Once a day 3. 3–6 times a week 4. Once or twice a week 5. None at all
	In the past 30 days, how often did your asthma symptoms (such as wheezing, coughing, shortness of breath, chest tightness or pain) wake you up at night or earlier than usual in the morning?	1. 4 or more nights a week 2. 2 or 3 nights 3. Once a week 4. Once or twice 5. None at all
	In the past 30 days, how often have you used a rescue inhaler, nebulizer treatment, or other controlling medication (such as albuterol)?	1. 3 or more times per day 2. 1 or 2 times per day 3. 2 or 3 times per week 4. Once a week or less 5. Not at all
	How would you rate your asthma control during the past 30 days?	1. Not controlled at all 2. Poorly controlled 3. Somewhat controlled 4. Well controlled 5. Completely controlled
**Uncontrolled asthma (ACT < = 19)**	Adults who have asthma control test value of fewer than 20	Yes/No
**Asthma diagnosis**	Has a doctor, nurse or other health professional **EVER** told you that you had any of the following lung or respiratory conditions?	Choose all that apply 1. COPD 2. Chronic Bronchitis 3. Emphysema 4. Asthma 5. Some other lung or respiratory condition 6. None of the above
	In the past 12 months, has a doctor ever told you that you had any of the following lung or respiratory conditions	Choose all that apply 1. COPD 2. Chronic Bronchitis 3. Emphysema 4. Asthma 5. Some other lung or respiratory condition 6. None of the above
**Tobacco use**		
**Current established cigar smokers**	Adult respondents who have smoked cigars fairly regularly and currently smoke every day or some days	Yes/No
**Current established traditional cigar smokers**	Adult respondents who have smoked traditional cigars fairly regularly and currently smoke every day or some days	Yes/No
**Current established cigarillo smokers**	Adult respondents who have smoked cigarillos fairly regularly and currently smoke every day or some days	Yes/No
**Current established filtered cigar smokers**	Adult respondents who have smoked filtered cigars fairly regularly and currently smoke every day or some days	Yes/No
**Current established cigarette smokers**	Adult respondents who smoked 100 + cigarettes in their lifetime and currently smoke every day or some days	Yes/No
**Pack-year history of cigarette smoking**	Adult number of cigarette packs smoked per day multiplied by the number of years they have smoked fairly regularly	Variable was Winsorized to the 95th percentile to limit the influence of outliers
**Other tobacco product use**	Adult respondents who are current established users of other tobacco products including ENDS, pipe or hookah, smokeless tobacco, or snus	Yes/No
**Adult Cigar and/or Cigarette Smoking Status (4 categories)**	1. Never user of cigarettes or cigars; 2. Current established cigar smokers and not a current established smoker of cigarettes; 3. Current established cigarette smoker and not a current established cigar smoker; 4. Current established smoker of both cigarettes and cigars. All categories could include users of other tobacco products	1 = Never cigar or cigarettes smokers 2 = Current established exclusive cigar smokers 3 = Current established exclusive cigarette smokers 4 = Current established dual smokers of cigars and cigarettes
**Adult Traditional Cigar and/or Cigarette Smoking Status (4 categories)**	1. Never user of cigarettes or traditional cigars; 2. Current established traditional cigar smokers and not a current established smoker of cigarettes; 3. Current established cigarette smoker and not a current established traditional cigar smoker; 4. Current established smoker of both cigarettes and traditional cigars. All categories could include users of other tobacco products	1 = Never traditional cigar or cigarettes smokers 2 = Current established exclusive traditional cigar smokers 3 = Current established exclusive cigarette smokers 4 = Current established dual smokers of traditional cigars and cigarettes
**Adult Cigarillo and/or Cigarette Smoking Status (4 categories)**	1. Never user of cigarettes or cigarillos; 2. Current established cigarillo smokers and not a current established smoker of cigarettes; 3. Current established cigarette smoker and not a current established cigarillo smoker; 4. Current established smoker of both cigarettes and cigarillos. All categories could include users of other tobacco products	1 = Never cigarillo or cigarette smokers 2 = Current established exclusive cigarillo smokers 3 = Current established exclusive cigarette smokers 4 = Current established dual smokers of cigarillos and cigarettes
**Adult Filtered Cigar and/or Cigarette Smoking Status (4 categories)**	1. Never user of cigarettes or filtered cigars; 2. Current established filtered cigar smokers and not a current established smoker of cigarettes; 3. Current established cigarette smoker and not a current established filtered cigar smoker; 4. Current established smoker of both cigarettes and filtered cigars. All categories could include users of other tobacco products	1 = Never filtered cigar or cigarettes smokers 2 = Current established exclusive filtered cigar smokers 3 = Current established exclusive cigarette smokers 4 = Current established dual smokers of filtered cigars and cigarettes
**Duration of cigar use**	Approximation of years of cigar use. If multiple cigar types were used, the maximum duration was taken	
**Other covariates**		
**Secondhand smoke exposure**	During the past seven days, about how many hours were you around others who were smoking [whether or not you were smoking yourself]? Include time in your home, in a car, at work, or outdoors	Variable was Winsorized to the 99th percentile to limit the influence of outliers
**Past month marijuana use**	Used marijuana within the past 30 days	Yes/No
**Use of asthma medications**	In the past 12 months, which of the following medications did you regularly take for asthma? 1 = Quick-relief inhaler—for example: albuterol (ProAir, Ventolin, Xopenex), ipratropium (Atrovent), or a combination inhaler (Combivent) 2 = Controller or long-acting inhaler including steroid inhaler – for example: beclomethasone (Qvar), fluticasone (Flovent), salmeterol (Serevent), tiotropium (Spiriva), or a combination inhaler (Advair) 3 = Other controlling medication – for example: montelukast (Singulair), zafirlukast (Accolate), theophylline, roflumilast (Daliresp) 5 = Oxygen therapy 6 = Some other asthma medication	1 = No medication use 2 = Quick relief only 3 = Control medications
**COPD or other non-asthma respiratory diseases**	Has a doctor, nurse or other health professional **EVER** told you that you had any of the following lung or respiratory conditions? Choose all that apply **1. COPD** **2. Chronic Bronchitis** **3. Emphysema** 4. Asthma 5. Some other lung or respiratory condition 6. None of the above	Yes/No
**Body Mass Index (BMI)**		1 = Underweight (BMI under 18.5) 2 = Normal (BMI 18.5–24.99) 3 = Overweight (BMI 25–29.99) 4 = Class 1 Obese (BMI 30–34.99) 5 = Class 2 Obese (BMI 35 +)
**Age-4 categories**		1 = 18–24 2 = 25–39 3 = 40–54 4 = 55 +
**Sex**		1 = Male 2 = Female
**Education-4 categories**		1 = Less than high school or GED 2 = High school graduate 3 = Some college or associate degree 4 = Bachelor’s or advanced

Abbreviations: PATH = Population Assessment of Tobacco and Health

^a^Respiratory symptom index developed based on responses to questions from the International Study of Asthma and Allergy in Children
