# Supplementary material for: Respiratory symptoms and outcomes among cigar smokers: findings from the Population Assessment of Tobacco and Health (PATH) study waves 2–5 (2014–2019)
Source: Respir Res. 2024 Apr 27;25:185. doi: 10.1186/s12931-024-02818-x (PMC11055341; doi:10.1186/s12931-024-02818-x)
Supplement: Supplementary file 1 — Additional file 1: Supplemental Table 1. Characteristics of adults at Wave 2 (2014-2015) by respiratory symptoms and outcomes. Supplemental Table 2a. Associations between tobacco use status and functionally important respiratory symptoms (Wave 2 - Wave 5 population averaged), PATH Study 2014-2019. Supplemental Table 2b. Associations between tobacco use status and respiratory outcomes, stratified by adults ages 18-39 and 40+ (Wave 2 – Wave 5 population averaged), PATH Study 2014-2019. Supplemental Table 3. Associations between cigar and cigarette use status at Wave 2 and new reports of ≥ 3 functionally important respiratory symptoms at Wave 3, Wave 4, or Wave 5, PATH Study 2014-2019. [file 12931_2024_2818_MOESM1_ESM.docx]

**Supplemental Table 1. Characteristics of adults at Wave 2 (2014-2015) by respiratory symptoms and outcomes**

| Measures | **Functionally important respiratory symptoms^1^  (≥ 3 symptoms)  N=1,635** | | | **Lifetime asthma diagnosis N=1,230** | | | **Uncontrolled asthma^2^ (ACT ≤ 19)  N=219** | | |
| --- | --- | --- | --- | --- | --- | --- | --- | --- | --- |
|  | Unweighted N | Weighted % or Mean | Weighted 95% CI | Unweighted N | Weighted % or Mean | Weighted 95% CI | Unweighted N | Weighted % or Mean | Weighted 95% CI |
| **Age** |  |  |  |  |  |  |  |  |  |
| 18-24 | 354 | 13.5 | 12.1, 15.1 | 485 | 24.8 | 22.3, 27.6 | 69 | 16.9 | 12.3, 22.6 |
| 25-39 | 552 | 34.3 | 31.6, 37.1 | 396 | 33.6 | 30.1, 37.4 | 64 | 22.9 | 17.0, 30.2 |
| 40-54 | 462 | 30.4 | 27.6, 33.3 | 235 | 25.4 | 22.4, 28.7 | 59 | 38.3 | 28.5, 49.1 |
| 55+ | 267 | 21.8 | 18.9, 24.9 | 114 | 16.1 | 12.6, 20.3 | 27 | 21.9 | 13.7, 33.1 |
| **Sex** |  |  |  |  |  |  |  |  |  |
| Male | 752 | 48.6 | 45.7, 51.5 | 467 | 36.9 | 33.3, 40.8 | 63 | 28.8 | 21.5, 37.5 |
| Female | 882 | 51.4 | 48.5, 54.3 | 763 | 63.1 | 59.2, 66.7 | 156 | 71.2 | 62.5, 78.5 |
| **Race** |  |  |  |  |  |  |  |  |  |
| Non-Hispanic white | 1,121 | 72.2 | 69.1, 75.0 | 674 | 59.6 | 55.5, 63.5 | 102 | 45.6 | 36.4, 55.0 |
| Non-Hispanic black | 210 | 11.7 | 9.8, 13.9 | 230 | 15.9 | 13.2, 18.9 | 52 | 21.1 | 14.6, 29.5 |
| Non-Hispanic other | 110 | 7.2 | 5.4, 9.4 | 102 | 8.9 | 6.8, 11.7 | 17 | 11.3† | 4.9, 23.9 |
| Hispanic | 171 | 9.0 | 7.4, 11.0 | 205 | 15.6 | 12.4, 19.4 | 39 | 22.0 | 13.8, 33.2 |
| **Education** |  |  |  |  |  |  |  |  |  |
| Less than high school or GED | 458 | 23.9 | 21.7, 26.1 | 277 | 17.3 | 14.9, 20.0 | 75 | 32.9 | 24.9, 42.1 |
| High school graduate | 394 | 23.5 | 20.7, 26.5 | 278 | 21.5 | 18.4, 25.1 | 56 | 23.8 | 17.1, 31.9 |
| Some college or associate degree | 593 | 33.9 | 30.8, 37.1 | 480 | 36.5 | 32.8, 40.4 | 74 | 35.9 | 27.2, 45.5 |
| Bachelor’s or advanced | 187 | 18.8 | 15.8, 22.2 | 192 | 24.6 | 21.1, 28.6 | 14 | 7.5† | 3.7, 14.6 |
| **BMI^3^** |  |  |  |  |  |  |  |  |  |
| Underweight | 44 | 2.1 | 1.4, 3.0 | 34 | 2.5 | 1.4, 4.3 | 8 | 3.8† | 1.5, 9.4 |
| Normal | 483 | 28.4 | 25.6, 31.5 | 399 | 31.1 | 27.3, 35.1 | 59 | 26.3 | 18.4, 36.1 |
| Overweight | 480 | 31.6 | 28.7, 34.7 | 340 | 31.4 | 27.4, 35.7 | 59 | 30.0 | 20.3, 41.8 |
| Class 1 obese | 314 | 20.0 | 17.9, 22.4 | 202 | 15.9 | 13.3, 19.1 | 33 | 15.0 | 10.1, 21.7 |
| Class 2+ obese | 286 | 17.9 | 15.5, 20.5 | 226 | 19.1 | 16.3, 22.3 | 59 | 24.9 | 17.7, 33.8 |
| **Cigar and cigarette use status** |  |  |  |  |  |  |  |  |  |
| Never cigar or cigarette smokers | 236 | 29.7 | 26.3, 33.5 | 492 | 60.8 | 57.3, 64.1 | 59 | 48.3 | 38.0, 58.7 |
| Current established exclusive cigar smokers | 51 | 2.3 | 1.7, 3.2 | 55 | 2.9 | 2.1, 3.8 | 10 | 2.6† | 1.2, 5.5 |
| Current established exclusive cigarette smokers | 1220 | 61.6 | 58.1, 65.0 | 600 | 32.5 | 29.5, 35.8 | 127 | 41.6 | 33.0, 50.6 |
| Current established dual smokers of cigars and cigarettes | 128 | 6.3 | 5.1, 7.7 | 83 | 3.8 | 2.9, 5.1 | 23 | 7.6 | 4.5, 12.5 |
|  |  |  |  |  |  |  |  |  |  |
| **Cigarette pack years (mean)^4^** | 1,595 | 14.4 | 13.1, 15.6 | 1,214 | 5.9 | 4.5, 7.2 | 216 | 8.7 | 5.8, 11.6 |
| **Duration of cigar use (mean)^5^** | 1,626 | 6.5 | 6.1, 7.0 | 1,227 | 2.8 | 2.4, 3.2 | 219 | 3.5 | 2.0, 5.0 |
| **Past month marijuana use** | 472 | 23.5 | 21.4, 25.8 | 254 | 13.3 | 11.3, 15.6 | 58 | 18.0 | 13.1, 24.2 |
| **Secondhand smoke exposure in hours (mean)^6^** | 1,616 | 15.7 | 14.1, 17.2 | 1,210 | 8.0 | 6.8, 9.1 | 219 | 10.2 | 7.2, 13.2 |
| **Other tobacco use (current established)^7^** | 272 | 12.8 | 11.2, 14.5 | 165 | 8.0 | 6.9, 9.3 | 32 | 9.6 | 6.4, 14.4 |

Notes: Percents are weighted using the Wave 5 all-waves longitudinal weights for the Wave 1 Cohort (R05_A_A01WGT). To be included in the analysis, respondents must have participated in all 5 waves and be in the analytic sample. Ns are unweighted.

† Estimate should be interpreted with extra caution because it has low statistical precision. It is based on a denominator sample size of less than 50, or the coefficient of variation of the estimate or its complement is larger than 30%.

^1^FIRS is a 9-item index (range 0-9, higher = worse):

Have you ever had wheezing or whistling in the chest at any time in the past?

Have you had wheezing or whistling in the chest in the past 12 months?

How many attacks of wheezing have you had in the past 12 months?

In the past 12 months, how often, on average has your sleep been disturbed due to wheezing?

In the past 12 months, has wheezing ever been severe enough to limit your speech to only one or two words between breaths?

In the past 12 months, has your chest sounded wheezy during or after exercise?

In the past 12 months, have you had a dry cough at night, apart from a cough associated with a cold or chest infection?

^2^ACT (Asthma Control Test) is a 5-item self-administered questionnaire (range 5-25, higher = better asthma control):

In the past 30 days, how much of the time did your asthma keep you from getting as much done at work, school or at home?

In the past 30 days, how often have you had shortness of breath?

In the past 30 days, how often did your asthma symptoms (such as wheezing, coughing, shortness of breath, chest tightness or pain) wake you up at night or earlier than usual in the morning?

In the past 30 days, how often have you used a rescue inhaler, nebulizer treatment, or other controlling medication (such as albuterol)?

How would you rate your asthma control during the past 30 days?

Among adults with asthma

^3^Underweight = BMI < 18.5

Normal = BMI 18.5 to < 25

Overweight = BMI 25 to < 30

Class 1 obese = BMI 30 to < 35

Class 2 obese = BMI 35+

^4^Adult number of cigarette packs smoked per day multiplied by the number of years they have smoked fairly regularly.

^5^Adult approximation of number of years smoked cigars. Respondents who used multiple products and did not know, refused to provide, or gave an improbable response to the number of years used for one or more products but gave a valid response to the number of years used for the other product had their values set based on their valid responses.

^6^During the past seven days, about how many hours were you around others who were smoking [whether or not you were smoking yourself]?

^7^Current established use of ENDS, pipe, hookah, smokeless tobacco /snus.

**Supplemental Table 2a. Associations between tobacco use status and functionally important respiratory symptoms (Wave 2 - Wave 5 population averaged), PATH Study 2014-2019**

| Tobacco use status^1^ | **Functionally important respiratory symptoms (≥ 3 symptoms) Obs=41,919^a^** | | | |
| --- | --- | --- | --- | --- |
|  | Obs | Weighted %  (95% CI) | Unadjusted OR (95% CI) | Adjusted OR  (95% CI) |
| Never smokers of cigarettes or cigars Obs=19,276 | 1,282 | 6.0 (5.3, 6.7) | **Ref** | **Ref** |
| Current established cigar smokers Obs=1,898 | 191 | 8.8 (7.4, 10.5) | **2.10 (1.62, 2.72)** | 1.27 (0.94, 1.71) |
| Current established cigarette smokers Obs=19,027 | 4,389 | 22.1 (21.1, 23.1) | **4.86 (4.19, 5.64)** | **2.48 (2.08, 2.97)** |
| Current established dual smokers of cigars and cigarettes Obs=1,718 | 424 | 24.4 (21.3, 27.7) | **5.14 (4.07, 6.48)** | **2.85 (2.11, 3.85)** |

Bolded estimates are statistically significant (p < 0.05).

Ns are unweighted; percentages and ORs are weighted using the Wave 5 all-waves weights for the Wave 1 Cohort.

OR=odds ratio; Obs=observations

^1^Current established any cigar smoking was defined as ever smoking any cigar fairly regularly and now smoking every day or some days.

Excludes those with COPD and other non-asthma respiratory diseases

Adjusted for age (18-24, 25-39, 40-54, 55+), sex, race/ethnicity, education, BMI, cigarette pack years, duration of cigar use, secondhand smoke exposure, past month marijuana use, and current established use of at least one of: ENDS, pipe, hookah, smokeless tobacco, snus.

^a^Overall Obs represents unadjusted model and does not take into account missingness on covariates. 11,339 observations were missing on covariates in the adjusted model.

**Supplemental Table 2b. Associations between tobacco use status and respiratory outcomes, stratified by adults ages 18-39 and 40+ (Wave 2 – Wave 5 population averaged), PATH Study 2014-2019**

| **Lifetime asthma diagnosis**  **18-39 Obs=28,284; 40+ Obs=15,425^a^** | | | | | **Uncontrolled asthma^1^ (ACT ≤ 19)**  **18-39 Obs=4,391; 40+ Obs=1,425^a^** | | | | |
| --- | --- | --- | --- | --- | --- | --- | --- | --- | --- |
| Tobacco use status^2^ | Obs | Weighted %  (95% CI) | Unadjusted OR (95% CI) | Adjusted OR  (95% CI) | Tobacco use status^2^ | Obs | Weighted %  (95% CI) | Unadjusted OR  (95% CI) | Adjusted OR (95% CI) |
| **Ages 18-39** |  |  |  |  | **Ages 18-39** |  |  |  |  |
| Never smokers of cigarettes or cigars  *Obs=15,385* | 2,631 | 14.1 (12.8, 15.4) | **Ref** | **Ref** | Never smokers of cigarettes or cigars  *Obs=2,433* | 220 | 7.6 (6.0, 9.7) | **Ref** | **Ref** |
| Current established cigar smokers  *Obs=1,138* | 195 | 15.2 (11.9,19.1) | 0.93 (0.80, 1.08) | 0.98 (0.84, 1.14) | Current established cigar smokers  *Obs=174* | 24 | 13.0 (7.4, 22.0) | 1.82 (0.57, 5.79) | 2.40 (0.52, 11.17) |
| Current established cigarette smokers  *Obs=10,602* | 1,753 | 14.2 (12.8, 15.6) | 1.02 (0.90, 1.15) | 1.02 (0.90, 1.17) | Current established cigarette smokers  *Obs=1,571* | 318 | 19.4 (16.2, 23.0) | **2.96 (1.97, 4.45)** | **2.00 (1.25, 3.22)** |
| Current established dual smokers of cigars and cigarettes  *Obs=1,159* | 232 | 17.2 (14.1, 20.8) | 1.03 (0.90, 1.16) | 1.02 (0.89, 1.16) | Current established dual smokers of cigars and cigarettes  *Obs=213* | 54 | 29.0 (20.3, 39.5) | **4.80 (2.59, 8.90)** | **3.27 (1.47, 7.28)** |
|  |  |  |  |  |  |  |  |  |  |
| **Ages 40+** |  |  |  |  | **Ages 40+** |  |  |  |  |
| Never smokers of cigarettes or cigars  *Obs=4,802* | 488 | 9.1 (7.7, 10.8) | **Ref** | **Ref** | Never smokers of cigarettes or cigars  *Obs=465* | 85 | 19.1 (14.0, 25.5) | **Ref** | **Ref** |
| Current established cigar smokers  *Obs=820* | 80 | 8.9 (6.0, 13.0) | 0.98 (0.75, 1.29) | 1.27 (0.84, 1.91) | Current established cigar smokers  *Obs=73* | 11 | 17.7† (8.6, 32.9) | 0.93 (0.34, 2.55) | 2.28 (0.59, 8.85) |
| Current established cigarette smokers  *Obs=9,156* | 875 | 9.4 (8.1, 10.8) | 0.99 (0.75, 1.30) | 1.16 (0.79, 1.70) | Current established cigarette smokers  *Obs=804* | 228 | 28.1 (22.6, 34.4) | 1.47 (0.89, 2.43) | 1.34 (0.53, 3.37) |
| Current established dual smokers of cigars and cigarettes  *Obs=647* | 89 | 12.0 (8.5, 16.7) | 1.03 (0.78, 1.36) | 1.33 (0.89, 1.98) | Current established dual smokers of cigars and cigarettes  *Obs=83* | 30 | 39.2 (25.9, 54.4) | **2.25 (1.12, 4.52)** | 2.91 (0.87, 9.68) |

† Estimate should be interpreted with extra caution because it has low statistical precision. It is based on a denominator sample size of less than 50, or the coefficient of variation of the estimate or its complement is larger than 30%.

^1^Among adults with an asthma diagnosis.

Bolded estimates are statistically significant (p < 0.05).

Ns are unweighted; percentages and ORs are weighted using the Wave 5 all-waves weights for the Wave 1 Cohort.

OR=odds ratio; Obs=observations

^2^Current established any cigar smoking was defined as ever smoking any cigar fairly regularly and now smoking every day or some days.

Excludes those with COPD and other non-asthma respiratory diseases

Adjusted for age (18-24, 25-39, 40-54, 55+), sex, race/ethnicity, education, BMI, cigarette pack years, duration of cigar use, secondhand smoke exposure, past month marijuana use, and current established use of at least one of: ENDS, pipe, hookah, smokeless tobacco, snus. Uncontrolled asthma model also adjusted for use of asthma medications.

^a^Overall Obs represents unadjusted model and does not take into account missingness on covariates. 7,061 observations were missing on covariates for the 18-39 ever asthma adjusted model; 3,126 for the 40+ ever asthma adjusted model; 1,399 for the 18-39 uncontrolled asthma adjusted model; 375 for the 40+ uncontrolled asthma adjusted model.

**Supplemental Table 3. Associations between cigar and cigarette use status at Wave 2 and new reports of ≥ 3 functionally important respiratory symptoms at Wave 3, Wave 4, or Wave 5, PATH Study 2014-2019**

| **Tobacco use status at Wave 2** | **Functionally important respiratory symptoms  (new reports of ≥ 3 symptoms at W3, W4, or W5) N = 7,210^a^** | | | |
| --- | --- | --- | --- | --- |
|  | N | Weighted %  (95% CI) | Unadjusted OR (95% CI) | Adjusted OR  (95% CI) |
| Never cigar or cigarette smokers (n=3,406) | 256 | 6.7 (5.8, 7.7) | **Ref** | **Ref** |
| Current established cigar smokers (n=347) | 44 | 12.7 (9.1, 17.5) | **2.03 (1.34, 3.09)** | **1.62 (1.02, 2.60)** |
| Current established cigarette smokers (n=3,171) | 765 | 22.7 (20.9, 24.6) | **4.09 (3.44, 4.87)** | **2.60 (2.05, 3.29)** |
| Current established dual smokers of cigars and cigarettes (n=286) | 74 | 23.9 (18.0, 31.1) | **4.39 (2.92, 6.58)** | **2.55 (1.57, 4.14)** |

Bolded estimates are statistically significant (p < 0.05)

Ns are unweighted; percentages and ORs are weighted using the Wave 5 all-waves weights for the Wave 1 Cohort.

OR=odds ratio

Excludes those with COPD and other non-asthma respiratory diseases.

Adjusted for age (18-24, 25-39, 40-54, 55+), sex, race/ethnicity, education, BMI, cigarette pack years, duration of cigar use, secondhand smoke exposure, past month marijuana use, and current established use of at least one of: ENDS, pipe, hookah, smokeless tobacco, snus.

^a^Overall N represents unadjusted column and does not take into account missingness on covariates. 583 observations were missing on covariates in the adjusted model.
